# Personal Health and Well-Being Determinants Associated with the Day-to-Day Variability of Sedentary Behaviour in Community-Dwelling People with Stroke

**DOI:** 10.3390/jcm14186560

**Published:** 2025-09-18

**Authors:** Lisa van Oirschot, Wendy Hendrickx, Martijn F. Pisters

**Affiliations:** 1 Clinical Health Sciences, Utrecht University Medical Center, Utrecht University, 3584 CS Utrecht, The Netherlands; 2 Department of Rehabilitation, Physiotherapy Science & Sport, UMC Utrecht Brain Center, Utrecht University, 3584 CS Utrecht, The Netherlands; w.hendrickx@fontys.nl; 3 Research Group Empowering Healthy Behaviour, Department of Health Innovations and Technology, Fontys University of Applied Sciences, 5612 MA Eindhoven, The Netherlands; 4 Center for Physical Therapy Research and Innovation in Primary Care, Julius Health Care Centers, 3454 PV De Meern, The Netherlands

**Keywords:** sedentary behaviour, health and well-being determinants, n-of-1 methods, stroke, personalized behavioural interventions

## Abstract

**Background:** To improve personalized behavioural interventions for people with stroke, it is crucial to understand which factors influence fluctuations in sedentary behaviour. This study aimed to explore the association between health and well-being determinants and daily variability in sedentary behaviour over time within community-dwelling people with stroke. **Methods:** An n-of-1 study design was conducted to examine the associations between determinants and sedentary behaviour during the RISE-intervention randomized multiple baseline study. The percentage of sedentary behaviour was measured daily with the ActivPAL activity monitor. The Visual Analogue Scale scores of the determinants were collected with diaries. Dynamic regression modelling (time-series analysis) was performed, starting with univariable and followed by multivariable linear regressions. **Results:** The analysis included twelve community-dwelling people with stroke (median age 65 years), with daily sedentary behaviour ranging from 55.5 to 81.4 percent. Objectively measured sleep length was positively associated with the percentage of sedentary behaviour in three participants (*p* = 0.001, *p* < 0.0001, and *p* = 0.045), and negatively associated in one participant (*p* = 0.002). Subjective sleep length (*p* = 0.016), fatigue (*p* = 0.013) and pain (*p* = 0.0098) were exclusively associated with the percentage of sedentary behaviour in one participant. No significant associations were found for sleep quality, happiness, stress or time pressure in five participants. **Conclusions:** The findings indicate inconsistent association patterns between health and well-being determinants and the day-to-day variability of sedentary behaviour across participants. This highlights the need for a personalized approach, as the determinants associated with the daily variability of sedentary behaviour in one individual may differ from those influencing another individual.

## 1. Introduction

Stroke is the second leading cause of death and the third leading cause of long-term disability in the world [1]. People with stroke are at higher risk for cardiovascular diseases including recurrent stroke [2]. There is also an association between high amounts of sedentary behaviour and cardiovascular diseases, especially when sedentary time is accumulated in prolonged bouts [3,4]. Sedentary behaviour is defined as follows: ‘Any waking behaviour characterized by an energy expenditure of 1.5 or less Metabolic Equivalent of Tasks (METs) while sitting, reclining, or lying’ [5]. People with stroke spend more time being sedentary, have longer uninterrupted sedentary bouts, and spend less time in moderate-to-vigorous physical activity compared to their healthy, age-matched peers [6,7,8,9]. Focusing on reducing sedentary behaviour might be a promising secondary prevention strategy for people with stroke who are highly sedentary [10,11]. Behavioural change interventions seem needed to support people with stroke, who are highly sedentary, to reduce and interrupt their sedentary behaviour [12].

To date, there is a lack of sustainable behavioural change interventions to reduce and interrupt sedentary behaviour in people with stroke who are highly sedentary [12]. Behaviour change techniques (e.g., self-monitoring, goal setting, action planning, and social support) for community-dwelling older adults are mainly focused on behaviour changes within an individual [13,14]. Yet, between-group approaches are the dominant analytical method, which looks for changes in sedentary behaviour between groups. However, between-group approaches cannot describe all the underlying individual factors for being sedentary [15]. Longitudinal within-individual designs, like n-of-1 studies, are based on the idea that an individual’s behaviour is more likely to occur when levels of relevant determinants are high than when they are low within the individual [16]. In an n-of-1 study design, repeated measurements are taken within a single participant to examine associations between fluctuations in influencing factors and changes in behaviour over time [17].

Individual sedentary behaviour trajectories may vary substantially [18]. A high degree of day-to-day variability in sedentary behaviour was noticed during the randomized multiple baseline study of the RISE-intervention. The RISE-intervention aims to Reduce and Interrupt Sedentary behaviour using a blended behavioural intervention to Empower people at risk towards sustainable 24 h movement behaviour changes [18]. It is essential to know potential health and well-being determinants that influence the degree of sedentary behaviour (e.g., to expand the knowledge that enriches an intervention protocol) [19]. Sleep length, sleep quality, happiness, fatigue, stress, time pressure and pain are health and well-being determinants that are related to cardiovascular diseases and may influence sedentary behaviour [20,21,22,23]. Prior group-level studies examining stable demographic- and stroke-related factors that are potentially associated with sedentary behavior showed that these factors explain only a small part of the between-person variability [6,24,25]. Therefore, this study targets fluctuating health and well-being determinants that may act as barriers to behaviour change. Poor sleep quality, time pressure, fatigue and pain are frequently named as barriers to increasing sedentary behaviour in other populations [19,26,27], and they are particularly relevant in people with stroke, where physical and cognitive limitations can further constrain behavioural adaption [28,29]. These health and well-being determinants are suitable for a quantitative study because these determinants can be measured with Ecological Momentary Assessments (EMAs). EMAs measure people’s current thoughts and experiences daily by using a diary with Visual Analogue Scales (VASs) [30]. Importantly, these aforementioned determinants are appropriate to include in a longitudinal within-individual design, because sleep length, sleep quality, happiness, fatigue, stress, time pressure and pain are determinants that can fluctuate from day to day [18]. A deeper understanding of the associations between these determinants and sedentary behaviour might explain the day-to-day variability of sedentary behaviour in people with stroke.

To date, the determinants that are associated with the day-to-day variability in sedentary behaviour remain inadequately understood. This study explores the association of health and well-being determinants (sleep length, sleep quality, happiness, fatigue, stress, time pressure and pain) with the variability in sedentary behaviour over time during the RISE-intervention within community-dwelling people with stroke.

## 2. Materials and Methods

### 2.1. Design

N-of-1 studies with longitudinal designs allow investigating the day-to-day variability within individuals and examine relationships between influencing determinants with sedentary behaviour over time [16]. Day-to-day repeated measures of determinants and sedentary behaviour were obtained from the RISE-intervention randomized multiple baseline study [18]. The RISE-intervention lasted 15 weeks and contained blended behavioural change interventions; a content overview is attached in Appendix A. Supplementary File S1—“RISE intervention details” provides comprehensive information about the weekly intervention schedule. Data were collected between February 2021 and May 2022. Informed consent was obtained from all participants. The RISE-intervention randomized baseline study was approved by the medical research ethics committee (NL73036.041.20).

### 2.2. Participants

In this secondary analysis, datasets were derived from participants involved in the RISE-intervention randomized multiple baseline study. The inclusion criteria were established to incorporate only datasets with less than 40 percent missing data, ensuring the robustness and reliability of the data. Fourteen participants were recruited through stroke units in hospitals or healthcare centers in the regions of Utrecht and Eindhoven in the Netherlands. The RISE-intervention targets community-dwelling people with stroke who are highly sedentary and were recently discharged from hospitals. These participants met the following criteria.

#### 2.2.1. Inclusion Criteria

To be eligible to participate in the randomized multiple baseline study of the RISE-intervention, a subject complied with the following criteria:

- Aged 18 years or older;
- Stroke diagnosed in hospital within past six months;
- People with stroke who have an increased sedentary behaviour pattern. Having a high amount of sedentary time (>9.5 h) and minimal number of one extra factor (>50 percent of the sedentary time is spent in bouts of more than 30 min and/or not complying with physical activity guidelines)
- Discharged to the home setting (or community-dwelling);
- Able to walk independently with or without walking aid, measured by a functional ambulation categories (FACs) score of at least three [31];
- Independent in activities of daily living pre-stroke, measured by a Barthel Index (BI) score of more than eighteen [32].

#### 2.2.2. Exclusion Criteria

A potential individual who met any of the following criteria was excluded from participation in the randomized multiple baseline study.

- Insufficient competency in the Dutch language to understand the RISE-intervention content;
- Incapable of understanding questionnaires and instructions, measured by a score of less than four on the Utrecht Communication Assessment (UCO);
- Severe comorbidities that prevent a participant from safely interrupting and reducing sedentary behaviour;
- Receiving physiotherapy in any other setting than primary care.

### 2.3. Sample Size

In n-of-1 studies, the statistical power is a function of the number of repeated measures in the individual participants, instead of the total number of participants [33]. To enable the detection of a pattern of associated determinants across individuals, a minimum of six to seven participants was necessary for this study [16,34,35,36]. The number of repeated measurements needed per participant was determined by combining the criteria of time-series analysis (a minimum of 50 repeated measures) and multiple regression analysis [33,37]. The number of needed repeated measures was 114, which was calculated with the formula N ≥ 50 + 8 × k (in which k is the number of independent variables, which were eight).

### 2.4. Measurements

Descriptive characteristics of the participants (age, gender, education level, comorbidities, living situation, alcohol consumption and aphasia) and their stroke (type, side and stroke classification utilizing the National Institutes of Health Stroke Scale) were obtained at baseline assessment by asking single questions.

Sedentary behaviour was operationalized as the percentage of waking hours spent sedentary and was measured each day throughout the intervention using the ActivPAL activity monitor (PAL Technologies Ltd., Glasgow, UK). The ActivPAL is a reliable (ICC 0.79–0.99) and valid (accuracy 98–100%) accelerometer that measures transitions between standing time and sedentary positions (sitting, reclining or lying) during waking hours [38,39,40]. This monitor was worn on the anterior side of the thigh by means of a waterproof skin-friendly tape and was replaced every two weeks due to maximum battery time. All ActivPAL data were assembled into one CSV file for one participant using a knitter program. Waking hours and sitting hours were identified by using the ProcessingPAL program [41]. The percentage of waking hours spent sedentary was calculated by dividing the total sitting hours by the waking hours and multiplying by one hundred.

Health and well-being determinants of each intervention day were obtained using diaries (the format is added in Appendix B, except for objectively measured sleep length, which was calculated by subtracting the waking hours (measured with the ActivPAL) from 24 h. The diaries contain Ecological Momentary Assessments (EMAs), which were measured with Visual Analogue Scales (VASs) from 0 to 100, with 0 representing the lowest degree and 100 representing the highest degree of presence [42]. Participants were asked to complete the diary every morning when they woke up. A physiotherapist delivering the RISE-intervention reminded participants to keep track of the diary and made sure that there were sufficient diary pages to fill in.

### 2.5. Analysis

Processed data from the ActivPAL and data from diaries were assembled in one SPSS data file for each participant. Descriptive characteristics were analyzed by mean or median scores (depending on the normality of the data distribution) for the percentage of sedentary behaviour and all health and well-being determinants. The following analyses were conducted in SPSSv27 for each participant separately.

#### 2.5.1. Missing Cases

If missing data in ActivPAL data and/or diaries data was minimal (<5%), then data were imputed using the adjacent averaging method. This method imputes the mean value of the three antecedents and the three subsequent days [35,43].

If missing data in ActivPAL data and/or diaries data was more than five percent and missing at random, then multiple imputations were used. Multiple imputations were conducted using the “AmeliaView” program (version 1.8.0.), which is a software package within R that is conceptualized to accurately impute time-series data [44]. This program generated five imputed datasets for each participant, which were then transported to SPSS for analysis [16,34,44]. Because it is recommended to combine the results of the analyses instead of pooling the five imputed datasets [45], the subsequent analyses were conducted on all five imputed datasets separately.

If missing data in ActivPAL data and/or diaries data were more than forty percent, then the dataset of the individual was excluded from analysis, due to poor reliability in results [46].

#### 2.5.2. Dynamic Regression Modelling

Dynamic regression modelling is well-suited for n-of-1 studies because it analyzes how factors and outcomes change over time within an individual. By accounting for time-related dependencies and delayed effects, it captures dynamic relationships and fluctuations, providing precise insights into what influences behaviour or symptoms in single person trials [47]. Dynamic regression modelling including time-series analysis was conducted to express and examine each individual’s sedentary behaviour over time [35]. This analysis was conducted by accurately following the Vieira procedure, which is a 10-step protocol for dynamic regression modelling in SPSS [33]. This protocol contains a procedure to identify autocorrelation and conduct linear regression analysis.

#### 2.5.3. Autocorrelation

Autocorrelation exists when the present measurement is influenced by the previous measurement [16,33,34] (e.g., today’s pain score is, at least partially, influenced by yesterday’s pain score). Autocorrelation needed to be corrected before linear regression analyses are conducted to confine type-1 or type-2 error probability [48]. This procedure was conducted in the longitudinal data series of the percentage of sedentary time and all health and well-being determinants for the first seven days [33,47,49]. This is because the past effect of both the outcome and the determinants could influence the current day [33].

#### 2.5.4. Linear Regressions

Linear regressions were performed to identify the association of health and well-being determinants with the percentage of sedentary overtime in each individual. The analyses were adjusted for autocorrelation, the day of the week (week or weekend day) and study day to account for the potential influence of time on the relationship between outcome and determinant [33]. Univariable regressions were conducted to identify determinant(s) associated with sedentary behaviour, as indicated by an effect size with a significance level below *p* < 0.05. In cases where multiple determinants were potentially associated, as indicated by an effect size with a significance level below *p* < 0.15, multivariable regressions were performed. Multivariable regressions allow for the examination of independent associations in situations where multiple determinants appear to be associated [50].

A stepwise strategy was used because of the substantial number of potential determinants and the interest in determining the most influential variables. Assumptions were checked and multicollinearity was avoided by removing one of the determinants when two determinants were highly correlated (r > 0.850).

## 3. Results

### 3.1. Descriptive Statistics

#### 3.1.1. Participant Characteristics

**Figure 1:**
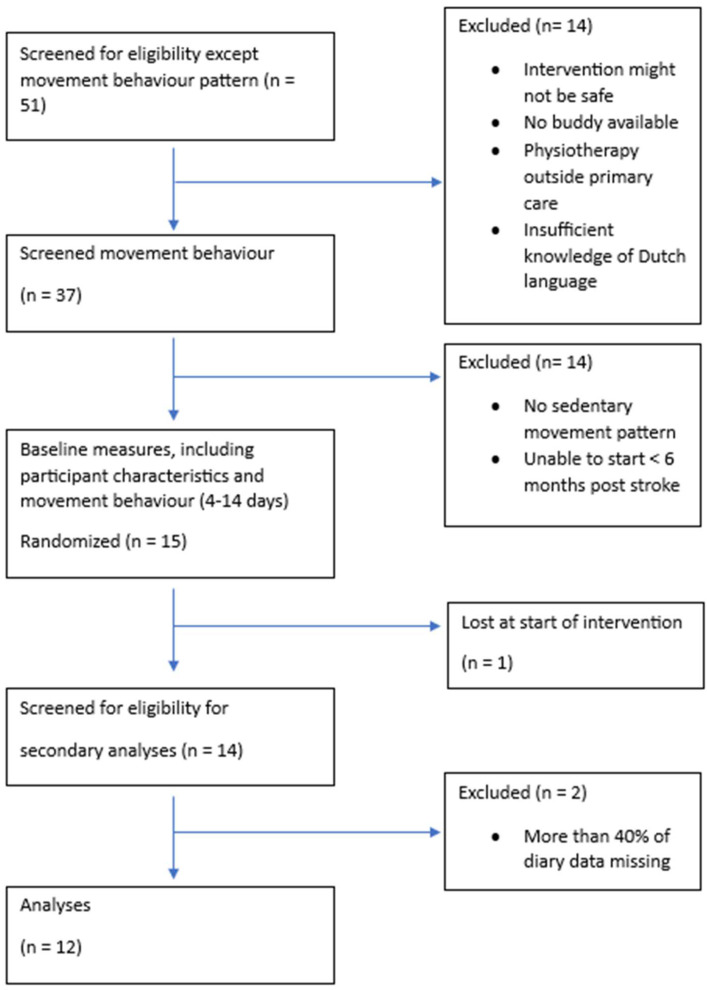
Flowchart participants.

Two of the fourteen participants were excluded because they had more than forty percent of missing data in their diaries. The final sample for this study included twelve participants of which three were females; see the flowchart in Figure 1. The age of the participants ranged from 49 to 78 years with a median of 65 (IQR 11.5). All participants had an ischaemic stroke, most in the left hemisphere (66.7%). An overview of all participants and stroke characteristics is displayed in Table 1.

#### 3.1.2. Days Measured, Missing Data and Imputation Strategies

The median number of days measured was 127. The amount of missing data ranged from 1.6 to 26.8 percent in the ActivPAL data, and from 0 to 32 percent in the diaries. Adjacent mean imputations were used for participants 1 and 8 because they had less than two percent of missing data in both the ActivPAL and diaries. Datasets from the other ten participants were imputed with AmeliaView. An overview of the intervention days, missing data and imputation strategy is displayed in Table 2.

#### 3.1.3. Descriptive Characteristics

The percentage of time spent sedentary during waking hours by the participants ranged from 55.5 to 81.4 with an overall median of 70.3 (IQR 10.8). Visualization of the data are included in Supplementary File S2. The overall medians show high levels of sleep quality (81.0) and happiness (90.0), and low levels of stress (10.0), time pressure (10.0) and pain (10.0). In six participants, the IQR was zero in sleep quality, happiness, time pressure, fatigue and pain. An IQR of 0 indicates that there is effectively no variation in the data, as the values of these determinants remained constant over time, resulting in a range of zero. This reflects low or no within-individual variability in the data series. The descriptive of the percentage of sedentary behaviour and determinants for each individual are represented in Table 3.

#### 3.1.4. Univariable Regression

The results of the univariable regression revealed a single significant association between objective sleep length and the percentage of sedentary behaviour in two participants (participant 1, *p* = 0.001; participant 10, *p* = 0.045). In five of the twelve participants, there was more than one determinant below the threshold of *p* < 0.15 (participants 3, 6, 8, 9 and 12). The following determinants for each of these five participants were selected for inclusion in the multivariable regressions. Objective sleep length (*p* = 0.076), sleep quality (*p* = 0.11) and time pressure (*p* = 0.11) in participant 3; fatigue (*p* = 0.013) and stress (*p* = 0.13) in participant 6; objective sleep length (*p* = 0.081) and pain (*p* = 0.010) in participant 8; objective sleep length (*p* = 0.005) and subjective sleep length (*p* = 0.10) in participant 9; and sleep quality (*p* = 0.13), fatigue (*p* = 0.072) and pain (*p* = 0.055) in participant 12. The effect sizes and corresponding *p*-values of each univariable regression are shown in Table 4.

#### 3.1.5. Multivariable Regressions

In three participants, the multivariable regression revealed a single significant association with the percentage of sedentary behaviour. Objective sleep length (*p* < 0.0001) in participant 3, fatigue (*p* = 0.013) in participant 6 and pain (*p* = 0.0098) in participant 12. In participant 9, objective (*p* = 0.002) and subjective sleep length (*p* = 0.016) were associated with the percentage of sedentary behaviour. In participant 8, no significant associations were found. The effect sizes and corresponding *p*-values of the multivariable regression of participants 3, 6, 8, 9 and 12 are shown in Table 5.

### 3.2. Determinants Associated with Sedentary Behaviour

**Figure 2:**
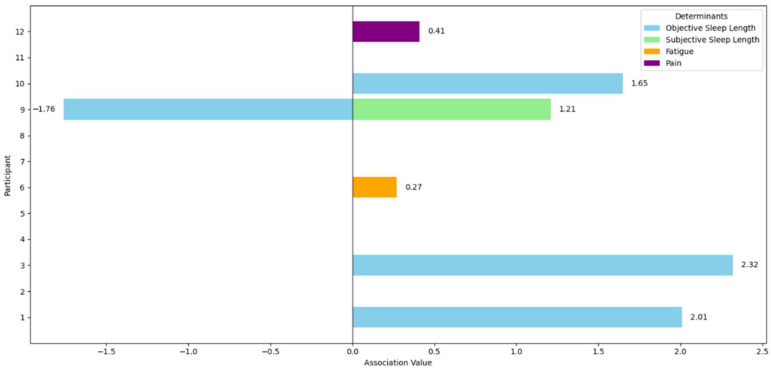
Associations between sedentary behaviour and health and well-being determinants across participants.

Objective sleep length was both positively associated (participants 1.3 and 10) and negatively associated (participant 9) with the percentage of sedentary behaviour. Subjective sleep length (participant 9), fatigue (participant 6) and pain (participant 12) were positively associated with the percentage of sedentary behaviour in their respective participants. Sleep quality, happiness, stress and time pressure showed no significant associations with the percentage of sedentary behaviour in all twelve participants. An overview of the single significant associations of the univariable regressions and the associated determinants of the multivariable regression is represented in Table 5. A visual overview of the diversity in associations across participants is shown in Figure 2. 

## 4. Discussion

The present study revealed an inconsistent pattern of associations between health and well-being determinants and the day-to-day variability of sedentary behaviour across participants. The inconsistencies observed—such as objectively measured sleep length (in three participants), subjectively measured sleep length (in one participant), fatigue (in one participant) and pain (in one participant), with no significant associations in the remaining participants—underscore the complex, individual-specific nature of factors influencing sedentary time after stroke. Sleep quality, happiness, stress and time pressure were not significantly associated with the percentage of sedentary behaviour in any of the twelve participants. All determinants, except for sleep length, exhibited low within-individual variability in most participants. The n-of-1 design allowed these inter-individual differences to become visible, supporting a person-centered approach to identifying health and well-being determinants associated with sedentary behaviour.

Previous n-of-1 studies examining the associations between potential determinants and movement behaviour over time have also identified inconsistent association patterns across participants [16,34,35,49,51]. The related studies also showed that n-of-1 studies can provide information about unique patterns and determinants of individual behaviour over time. They suggest that interventions were effective for some individuals but not for others, indicating that the impact of determinants of sedentary behaviour varies between individuals. This emphasizes that a determinant associated with the day-to-day variability of sedentary behaviour in one individual may not serve as an associated determinant for another individual. This is also in line with earlier studies showing that demographic and stroke-related factors explain only a small part of the between-person variance [6,25] and that considering other factors and adopting a more individualized behavioral approach seems appropriate. Consequently, when developing or implementing interventions aimed at reducing sedentary behaviour in individuals who had experienced a stroke, a ‘one-size-fits-all’ approach for taking health and well-being determinants into account seems to be inappropriate.

Conflicting results regarding the associations between sedentary behaviour and sleep length were found in a recent systematic review [52]. This aligns with the inconsistent associations observed for sleep length in the current study, suggesting that both insufficient and excessive sleep duration may influence sedentary behaviour on the following day [53,54]. These results indicate that certain health and well-being determinants could have both positive and negative associations with the variability of sedentary behaviour. However, these results should be interpreted with caution, as n-of-1 studies can identify patterns but not establish causal relationships [17].

Prior to conducting n-of-1 methods, it is crucial to make well-considered decisions to ensure sufficient variability in the data series [55]. In the analysis of the present study, no associations were identified between the determinants of sleep quality, happiness, stress, and time pressure and the day-to-day variability of sedentary behaviour. Most participants reported minimal to no issues with these determinants, which likely resulted in reduced fluctuations and consequently limited the within-individual variability of these determinants. In contrast, a related n-of-1 study found associations between sleep quality, happiness, stress and time pressure with physical activity in people during the retirement transition [35]. If sufficient within-individual variability for the determinants had been present in this study, as observed in the related study, associations for these determinants may have also been detected among community-dwelling people with stroke. Future research should involve follow-up n-of-1 studies employing purposive sampling to target individuals facing varied challenges with these determinants, thereby facilitating a more extensive examination of within-individual variability and its associations.

### 4.1. Strengths and Limitations

This is the first n-of-1 study that examined the association of health and well-being determinants with the variability of sedentary behaviour over time within community-dwelling people with stroke. Using dynamic regression modelling involving time-series analysis is a strength of this study. Involving time factors such as autocorrelation, weekdays and time trends is essential to create a draw. It ensures more reliable estimated coefficients and isolates the true associations of health and well-being determinants with the percentage of sedentary behaviour. Another strength was the sample size, which was relatively large compared to related n-of-1 studies [16,34,56]. This increased the power to detect association patterns across participants.

The high level of missing data within the data series of the ActivPAL and diaries in some participants was a limitation of this study. Missing data in the time-series data can be problematic because it disrupts the continuity, which influences the autocorrelation structure [33]. To tackle the missing data in the current study, the data were imputed by using AmeliaView. This program is superior in imputing time-series data because it takes the dynamics over time into account when imputing the data [44,49]. A second limitation is the lack of within-individual variability in the data series of multiple determinants. This limited the ability to find associations of health and well-being determinants with sedentary behaviour [55]. Lastly, the diaries were intended to be completed every day in the morning, though it might be possible that participants sometimes forgot and filled them out retrospectively, which could lead to recall bias.

### 4.2. Practical Implications

Considering the potential involvement of multiple health and well-being determinants in sedentary behaviour, a multidisciplinary approach may support a more comprehensive assessment and management of these factors in stroke rehabilitation. It is essential that both healthcare professionals and individuals with stroke are aware of the substantial inter-individual variability in the health and well-being determinants and their associations with variance in sedentary time. Such awareness can foster more accurate identification of relevant contributing factors, support shared decision-making and guide the development of personalized intervention strategies rather than the application of uniform protocols.

## 5. Conclusions

The findings of the present n-of-1 study highlight the inter-individual differences in the associations between health and well-being determinants and the variability of sedentary behaviour over time in community-dwelling people with stroke. This emphasizes the necessity of a personalized approach.

## Figures and Tables

**Table 1:** Participant characteristics of the twelve participants.

	1	2	3	4	5	6	7	8	9	10	11	12
Age (years)	68	65	65	69	65	62	77	54	78	49	55	69
Gender (male/female)	M	F	M	M	M	M	M	F	F	M	M	M
Education level (low/mid/high)	Mid	Low	High	Low	Mid	Mid	High	Mid	Mid	Low	Mid	High
Number of comorbidities	4	1	2	1	2	4	2	4	4	4	2	0
Living with spouse	Yes	Yes	Yes	Yes	Yes	No	Yes	No	No	Yes	Yes	Yes
Smoking	Yes	Yes	No	No	Yes	No	No	No	No	No	No	No
Alcohol (times a week)	1–2	1–2	1–2	1–2	1–2	1–2	1–2	1–2	0	1–2	1–2	0
Aphasia	No	Yes	No	No	Yes	No	No	No	No	Yes	Yes	No
Type of stroke (ischaemic or haemorrhagic)	I	I	I	I	I	I	I	I	I	I	I	I
Side of stroke (left, right or central)	L	L	R	R	L	C	L	L	L	R	L	L
Stroke classification NIHSS	3	8	0	1	NR	1	2	0	3	0	2	0

M = Male, F = Female; I = Ischaemic; L = Left, R = Right; C = Central; NR = Not Registered.

**Table 2:** Days measured, missing data and imputation strategy for each participant.

	Days Measured (*n*)	Missing Data in ActivPAL (%)	Missing Data in Diaries (%)	Imputation Strategy
1	124	1.6	<1.0	Adjacent mean imputation
2	124	11.3	32.0	AmeliaView
3	137	10.2	0.0	AmeliaView
4	118	12.6	9.2	AmeliaView
5	139	7.2	<1.0	AmeliaView
6	131	12.2	<1.0	AmeliaView
7	118	5.1	17.8	AmeliaView
8	130	1.5	0.0	Adjacent mean imputation
9	121	6.6	14.5	AmeliaView
10	142	26.8	0.0	AmeliaView
11	117	6.8	<1.0	AmeliaView
12	131	16.0	1.5	AmeliaView

**Table 3:** Descriptive characteristics of the percentage sedentary behaviour and health and well-being determinants for each participant.

	%SB ^a^	Objective Sleeping Time, Hours ^a^	Subjective Sleeping Time, Hours ^a^	Sleep Quality ^a^	Happiness ^a^	Fatigue ^a^	Stress ^a^	Time Pressure ^a^	Pain ^a^
1	**74.5** **(7.5)**	**8.0** **(1.2)**	**7.0** **(1.5)**	**60.0** **(30.0)**	**80.0** **(10.0)**	**50.0** **(10.0)**	10.0 (0.0)	10.0 (0.0)	10.0 (0.0)
2	**81.4** **(7.7)**	**8.8** **(1.6)**	**9.0** **(1.3)**	**82.0** **(15.1)**	**83.7** **(10.0)**	**30.6** **(30.0)**	**20.0** **(21.9)**	**11.1** **(14.1)**	**12.6** **(11.3)**
3	**69.4** **(15.9)**	**7.9** **(3.3)**	**6.0** **(1.0)**	90.0 (0.0)	90.0 (0.0)	10.0 (0.0)	10.0 (0.0)	10.0 (0.0)	10.0 (0.0)
4	**69.4** **(9.4)**	**9.7** **(0.9)**	**8.0** **(0.2)**	70.0 (0.0)	90.0 (0.0)	30.0 (0.0)	0.0 (0.0)	0.0 (0.0)	30.0 (0.0)
5	**55.5** **(22.7)**	**9.9** **(1.4)**	**7.0** **(2.0)**	**80.0** **(30.0)**	90.0 (0.0)	**60.0** **(30.0)**	**5.0** **(5.0)**	5.0 (0.0)	**60.0** **(10.0)**
6	**71.2** **(12.1)**	**8.9** **(1.5)**	**9.0** **(0.8)**	90.0 (0.0)	90.0 (0.0)	10.0 (0.0)	10.0 (0.0)	10.0 (0.0)	10.0 (0.0)
7	**79.4** **(7.0)**	**10.2** **(1.0)**	**10.0** **(1.0)**	100.0 (0.0)	100.0 (0.0)	0.0 (0.0)	0.0 (0.0)	0.0 (0.0)	0.0 (0.0)
8	**76.7** **(16.2)**	**10.4** **(1.5)**	8.0 (0.0)	80.0 (0.0)	80.0 (0.0)	50.0 (0.0)	20.0 (0.0)	20.0 (0.0)	50.0 (0.0)
9	**62.0** **(15.0)**	**8.9** **(2.8)**	**8.0** **(1.4)**	**99.00** **(1.6)**	99.0 (0.0)	**0.0** **(0.1)**	0.0 (0.0)	0.0 (0.0)	**0.0** **(10.4)**
10	**67.0** **(20.8)**	**9.1** **(2.2)**	**8.0** **(0.6)**	90.0 (0.0)	90.0 (0.0)	0.0 (0.0)	0.0 (0.0)	0.0 (0.0)	0.0 (0.0)
11	**61.3** **(19.8)**	**8.1** **(1.7)**	**8.0** **(1.0)**	50.0 (0.0)	50.0 (0.0)	50.0 (0.0)	**30.0** **(5.0)**	**25.0** **(10.0)**	0.0 (0.0)
12	**71.4** **(11.0)**	**8.1** **(0.9)**	**8.0** **(0.8)**	**70.0** **(10.0)**	80.0 (0.0)	**27.0** **(5.0)**	**20.0** **(5.0)**	**25.0** **(5.0)**	**25.0** **(5.0)**
Overall	70.3	9.0	8.0	81.0	90.0	28.8	10.0	10.0	10.0

^a^ Median (Interquartile range); SB = sedentary behaviour. Bolded values indicate an interquartile range that is larger than 0.

**Table 4:** Univariable regressions between each determinant and the percentage of sedentary time.

	The Percentage of Sedentary Behaviour							
	Objective Measured Sleep Length ^a^	Subjective Measured Sleep Length ^a^	Sleep Quality ^a^	Happiness ^a^	Fatigue ^a^	Stress ^a^	Time Pressure ^a^	Pain ^a^
1	**2.01** **(0.001)** *	0.12 (0.85)	−0.04 (0.35)	−0.036 (0.75)	−0.070 (0.20)	NA	NA	NA
2	−0.32 (0.52)	0.24 (0.55)	−0.026 (0.46)	0.028 (0.56)	0.026 (0.41)	0.013 (0.63)	0.0072 (0.72)	0.033 (0.43)
3	**1.25** (0.076) **	0.86 (0.37)	−0.24 (0.11) **	0.042 (0.67)	0.10 (0.23)	0.0005 (0.18)	**0.24** (0.11) **	NA
4	−0.20 (0.70)	−0.90 (0.42)	−0.11 (0.18)	−0.044 (0.39)	−0.052 (0.68)	−0.52 (0.56)	−1.84 (0.48)	0.0012 (0.45)
5	−1.93 (0.060) **	−0.31 (0.51)	−0.021 (0.58)	0.052 (0.73)	0.026 (0.57)	0.18 (0.36)	−0.20 (0.57)	−0.14 (0.23)
6	0.76 (0.25)	−0.50 (0.60)	−0.079 (0.65)	−0.40 (0.83)	**0.28** (0.013) *	**0.31** (0.13) **	NA	2.038 (0.44)
7	−0.97 (0.070) **	−1.021 (0.29)	−0.0068 (0.57)	0.053 (0.60)	0.15 (0.51)	0.15 (0.51)	NA	NA
8	**−1.62** (0.081) **	−1.74 (0.44)	−0.17 (0.54)	−0.26 (0.71)	−0.026 (0.95)	0.28 (0.41)	0.30 (0.45)	**−0.71** (0.010) *
9	**−1.51** (0.005) *	**1.05** (0.10) *	0.050 (0.56)	−0.004 (0.85)	0.035 (0.49)	0.004 (0.65)	0.043 (0.51)	−0.078 (0.47)
10	**1.65** (0.045) *	2.365 (0.32)	−0.21 (0.64)	−0.194 (0.20)	0.051 (0.65)	NA	NA	NA
11	0.85 (0.25)	1.22 (0.32)	0.08 (0.53)	0.23 (0.70)	−0.012 (0.91)	−0.036 (0.83)	−0.001 (0.78)	−0.021 (0.77)
12	−1.15 (0.37)	−1.03 (0.56)	−0.44 (0.13) **	0.744 (0.30)	**0.20** (0.072) **	0.118 (0.57)	0.161 (0.58)	**0.34** (0.055) **

^a^ effect size (*p*-value), * *p* < 0.05, ** *p* < 0.15; NA: Not available, regression was not conducted due to no variability in the data series. Bolded values indicate statistical significance.

**Table 5:** Multivariable regression between each determinant and the percentage of sedentary behaviour.

	The Percentage of Sedentary Behaviour							
	Objective Measured Sleep Length ^a^	Subjective Measured Sleep Length ^a^	Sleep Quality ^a^	Happiness ^a^	Fatigue ^a^	Stress ^a^	Time Pressure ^a^	Pain ^a^
1	**2.01** (0.001) *	-	-	-	-	-	-	-
3	**2.32** (<0.0001) *	-	−0.44 (0.15)	-	-	-	0.34 (0.19)	-
6	-	-	-	-	**0.27** (0.013) *	0.018 (0.86)	-	-
8	−0.143 (0.091)	-	-	-	-	-	-	−0.143 (0.088)
9	**−1.76** (0.002) *	**1.21** (0.016) *	-	-	-	-	-	-
10	**1.65** (0.045) *	**-**	-	-	-	-	-	-
12	-	-	−0.085 (0.48)	-	0.093 (0.32)	-	-	**0.41** (0.0098) *

^a^ effect size (*p*-value), * *p* < 0.05. Note: participants 1 and 10 were not conducted in multivariable analysis because of one significant association in the univariable regression; multivariable regression would have the same analysis. These participants were included in this table for the overview of all significant associations. Bolded values indicate statistical significance.

**Figure A1:**
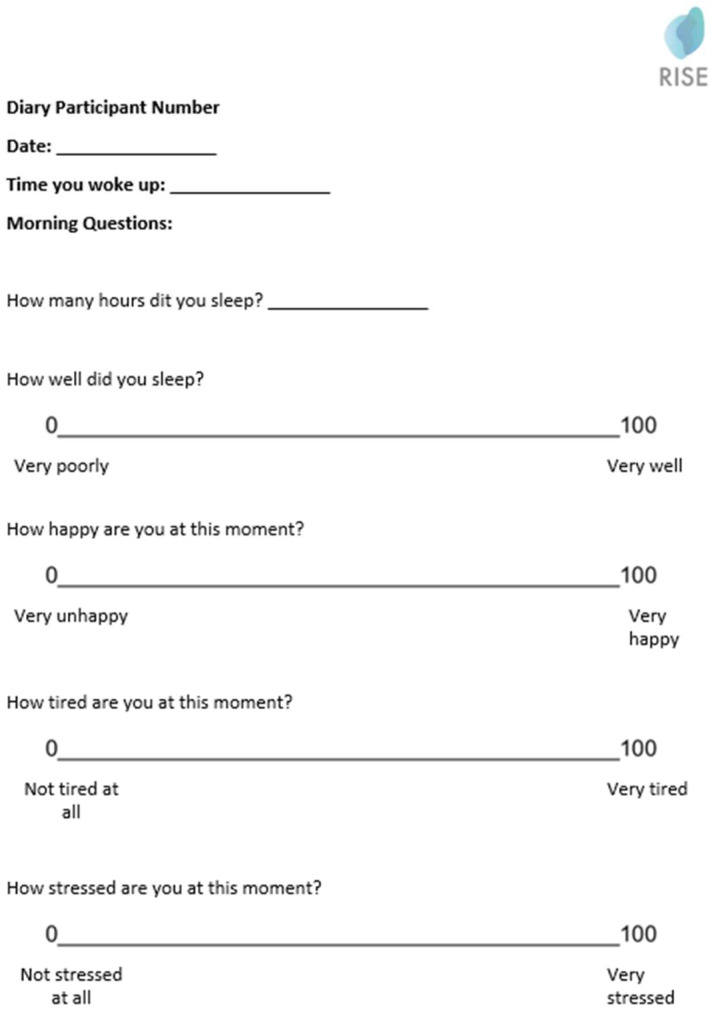
Health and well-being determinants form.

**Figure A2:**
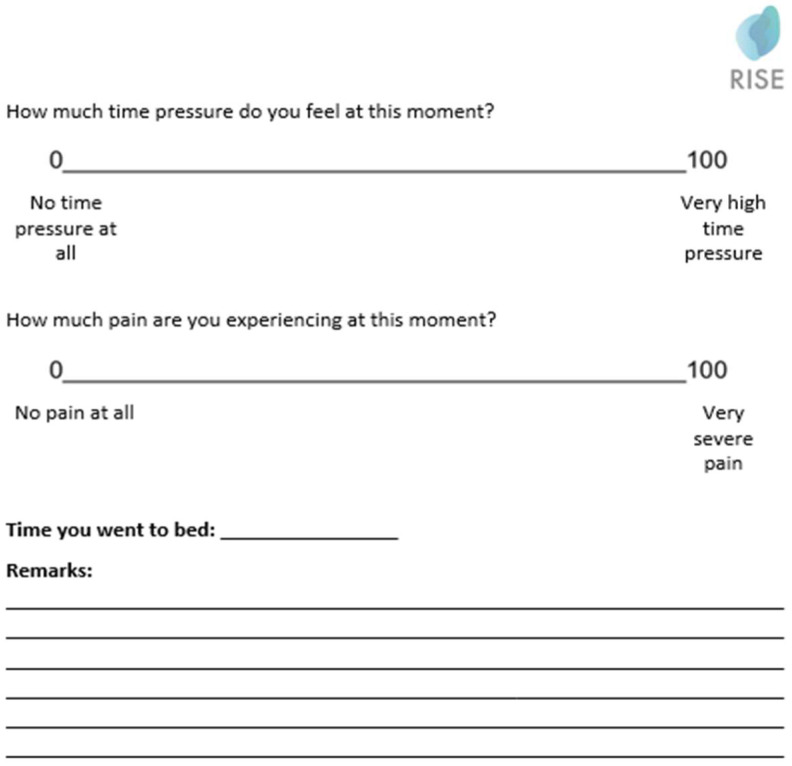
Health and well-being determinants form (continuation).

## Data Availability

The data presented in this study were derived from the RISE-intervention randomized multiple baseline study and are available upon reasonable request from the corresponding author.

## Supplementary Materials

The following supporting information can be download at https://www.mdpi.com/article/10.3390/jcm14186560/s1, File S1: RISE intervention details; File S2: Data visualization and Table PEM Physical activity.

## Abbreviations

The following abbreviations are used in this manuscript:

RISE	Reduce and Interrupt Sedentary behaviour using a blended behaviour intervention to Empower people at risk towards sustainable movement behaviour change.
METs	Metabolic Equivalent of Task
EMAs	Ecological Momentary Assessments
VAS	Visual Analogue Scale
FAC	Functional Ambulation Classification
BI	Barthel Index
UCO	Utrecht Coping List
PAL	Physical Activity Level
CSV	Comma-Separated Values
SPSS	Statistic Package for the Social Sciences
IQR	Interquartile Range

## Appendix A

### RISE-Intervention Protocol

All participants in this study received the RISE-intervention with or without participatory support (participating with a supportive buddy in their environment). The aim of the RISE-intervention is to support movement behaviour change, i.e., to reduce and interrupt sedentary behaviour in people with stroke who are highly sedentary [10]. The RISE-intervention is a behavioural change intervention that combines face-to-face physiotherapy sessions with eCoaching and self-monitoring. The content of the RISE-intervention is described in detail below.

The RISE-Intervention is personalized to the individual. Goalsetting, action planning, coping and setbacks will be discussed during the RISE-intervention.

**Table A1:** Weekly overview of the RISE-intervention.

Week	Mode of Delivery	Topic Covered
1	Face-to-face (meeting 1)	Introduction of the intervention
	eCoaching	Benefits of reducing sedentary behavior (1)
2	Face-to-face (meeting 2)	Behavioral diagnosis
	eCoaching	Benefits of reducing sedentary behavior (2)
3	Face-to-face (meeting 3)	Self-monitoring
	eCoaching	What is self-monitoring
4	Face-to-face (meeting 4)	Action planning and goal setting
	eCoaching	Prompts and cues
5	Face-to-face (meeting 5)	Building social support
	eCoaching	Maintenance task self-efficacy
6	Face-to-face (meeting 6)	Increasing self-confidence for reducing sedentary behavior
7	eCoaching	Review own behavior and the social and physical environment
8	Face-to-face (meeting 7)	Introduction setbacks
9	eCoaching	Habit formation and coping planning
10	Face-to-face (meeting 8)	Habit formation and setbacks
11	eCoaching	Sustainable change
12	Face-to-face (meeting 9)	Self-monitoring
13–14	eCoaching	Specific action and coping plan for the future
15	Face-to-face (meeting 10)	Future proof
